# Genetic Variations in the P2X7 Receptor: Opportunities and Challenges for Drug Development

**DOI:** 10.3390/ijms262110265

**Published:** 2025-10-22

**Authors:** Justin S. Y. Cheah, Kristen K. Skarratt, Stephen J. Fuller, Thomas Balle

**Affiliations:** 1 Sydney Pharmacy School, Faculty of Medicine and Health, The University of Sydney, Sydney, NSW 2006, Australia; jche8871@uni.sydney.edu.au; 2 Brain and Mind Centre, The University of Sydney, Camperdown, NSW 2050, Australia; 3 Sydney Medical School Nepean, Faculty of Medicine and Health, The University of Sydney, Nepean Hospital, Penrith, NSW 2750, Australia

**Keywords:** P2X7 receptor, single nucleotide polymorphism, haplotype, alternative splicing, extracellular signaling, allosteric modulation

## Abstract

The P2X7 receptor (P2X7R) is a ligand-gated, non-selective cation channel activated by extracellular ATP, a key danger signal in the cellular stress response. Due to its roles in inflammation and neurological disorders, it is an attractive therapeutic target. However, clinical trials of P2X7R antagonists have failed to show clinical efficacy. This review explores whether receptor polymorphisms, alternative splicing, and membrane composition contribute to these clinical trial failures. Genotyping of trial participants is highly recommended prior to enrolment, and in vitro functional studies should be wary of the membrane composition of cells expressing P2X7R. While the P2X7R shows promising therapeutic potential, there remain large gaps in research particularly in characterising haplotypes and alternatively spliced hetero- or homotrimers of the receptor. This results in the development and testing of agents without considering the genetic variability of the receptor, which we propose to be a large contributor to the lack of clinical success. We also summarise characteristics of the receptor and recent structural findings to discuss how computational approaches may help overcome these challenges of variability. Precision targeting of the receptor in disease states is warranted, and a collaborative approach covering multiple facets of the receptor will facilitate this.

## 1. Introduction

ATP is a key molecule in living cells, serving as an energy source for numerous enzymatic processes, including RNA and DNA synthesis. Although ATP primarily exists intracellularly, it has been established that ATP and its metabolites also play important roles in extracellular signalling. Cell death or disruption to cell membrane integrity leads to the release of significant amounts of ATP into the extracellular space, elevating its concentration above normal physiological levels. In this context, extracellular ATP acts as a danger signal, or “damage-associated molecular pattern” (DAMP) [1]. ATP, therefore, serves a dual role, first as an essential intracellular energy source and secondly as a potent extracellular signalling molecule during cellular stress or damage.

Extracellular ATP is detected by the transmembrane purinergic P2 receptors, which can be subdivided into two classes: P2X and P2Y. P2X receptors are trimeric, ligand-gated cation channels whereas P2Y receptors are G-protein coupled receptors. A subtype of the P2X receptors, the P2X7 receptor (P2X7R), is a key responder to ATP acting as a DAMP. The P2X7R is expressed in most immune cells and cells present in the central nervous system such as astrocytes and microglia [2]. This expression pattern, combined with its role in sensing cellular stress and the production of inflammatory cytokines including interleukin-1β (IL-1β) [3,4] and IL-18 [5,6], places the P2X7R at the centre of inflammation and disease processes. The receptor is well established as a driver of NOD-, LRR-, and pyrin domain-containing protein 3 (NLRP3) inflammasome activation and has been reported to interact with other membrane proteins and complexes, including pannexin-1, caveolin 1, lipid rafts, and transmembrane protein 16F (TMEM16F) [7]. Research has been focused on the P2X7R due to its involvement in inflammation, cancers, as well as neurological and immune system disorders [2,8,9,10,11,12,13]. For example, the P2X7R has been implicated in affecting the severity of coronavirus disease 2019 (COVID-19) [14]. A study found increased levels of the P2X7R in the plasma of COVID-19 patients which was positively correlated with disease severity [15], and a systematic review investigating the involvement of purinergic signalling in the treatment of COVID-19 suggested the P2X7R as a potential therapeutic target [16].

The P2X7R can function as a pattern recognition receptor that detects extracellular ATP as a DAMP. Of all the purinergic receptors, it has the lowest affinity to ATP and requires activation by elevated ATP levels [10]. Another defining feature is its ability to open a non-selective pore distinct from regular channel function, allowing for the flux of larger molecules and resulting in eventual cell death [11,17]. In this review, we have used the standard P2X7R nomenclature to refer to “channel function” as cation flux across the membrane, and “pore function” as the flux of larger molecules. It is important to distinguish between these terms to avoid confusion, and we advise against using “pore” to describe channel function. Although initially described for the P2X7R, other subtypes (including P2X2 and P2X4) have also shown pore-forming characteristics [18]. The mechanism of pore formation remains controversial and will only be discussed briefly. Significantly, unlike other P2X family members, the P2X7R does not typically exhibit desensitisation under sustained ATP exposure, which is the gradual reduction in inward current during sustained or repeated ATP application [17].

In vitro studies show that P2X7R antagonism blocks the release of inflammatory cytokines from immune cells [5,19,20]. This can lead to downstream effects such as reducing tumour growth [21,22,23], decreasing osteoclast formation [24], and preventing neuron degeneration [25]. In animal models of disease, antagonists show promise as mediators of neuropathic pain or inflammation [3,26,27,28,29,30]. Considering its involvement in inflammatory pathways and associations with various disease states, the P2X7R has become an attractive target for drug development and remains an active area of research [31,32]. However, in human clinical trials, P2X7R antagonists have failed to meet efficacy endpoints, and it is intriguing to ask why that could be. Multiple contributing factors are likely to be responsible for this failure. Ion channels often exhibit distinct functional outcomes depending on which inhibitory site is targeted, differences that are frequently influenced by the channel’s conformational state (open or closed) [33], and the structural specificity of the binding site [34,35,36]. In the case of the P2X7R, targeting orthosteric, allosteric, or modulatory sites can result in varied effects, including full channel blockade, selective inhibition of pore formation, modulation of cytokine release, and altered receptor sensitivity.

Clinical translation is also complicated due to species-specific differences in pharmacology and binding sites. Furthermore, the receptor possesses a high degree of genetic variations [37] such as single-nucleotide polymorphisms (SNPs) and alternative splicing, all of which can impact receptor structure and drug responsiveness. Genetic variations may ultimately result in altered protein function or surface expression. In this review, the structure of the P2X7R and its gating mechanism will be described including key ATP-binding residues, followed by an in-depth analysis of functionally relevant *P2RX7* SNPs and haplotypes. A focused review on alternative splicing, which can substantially alter P2X7R structure and function, has been recently conducted [38] and will also be discussed in this review. The aim of this review is to shed light on the impact of mutated states of P2X7Rs. Additionally, breakthroughs in structural biology have enabled the structure of an almost complete rat P2X7R (rP2X7R) to be determined by cryogenic electron microscopy (cryo-EM) [39,40,41]. Most recently, the human P2X7R (hP2X7R) has also been resolved in both the open and closed states [31]. Together with previous and ongoing site-directed mutagenesis studies, these studies provide mechanistic insights that will assist in drug discovery and provide the basis for a personalised medicine approach, enabling customised treatment for patients based on their P2X7 genetic makeup and the presence of alternatively spliced subunits.

## 2. Structural Insights and Gating Mechanisms

### 2.1. Subunit Structure

**Figure 1:**
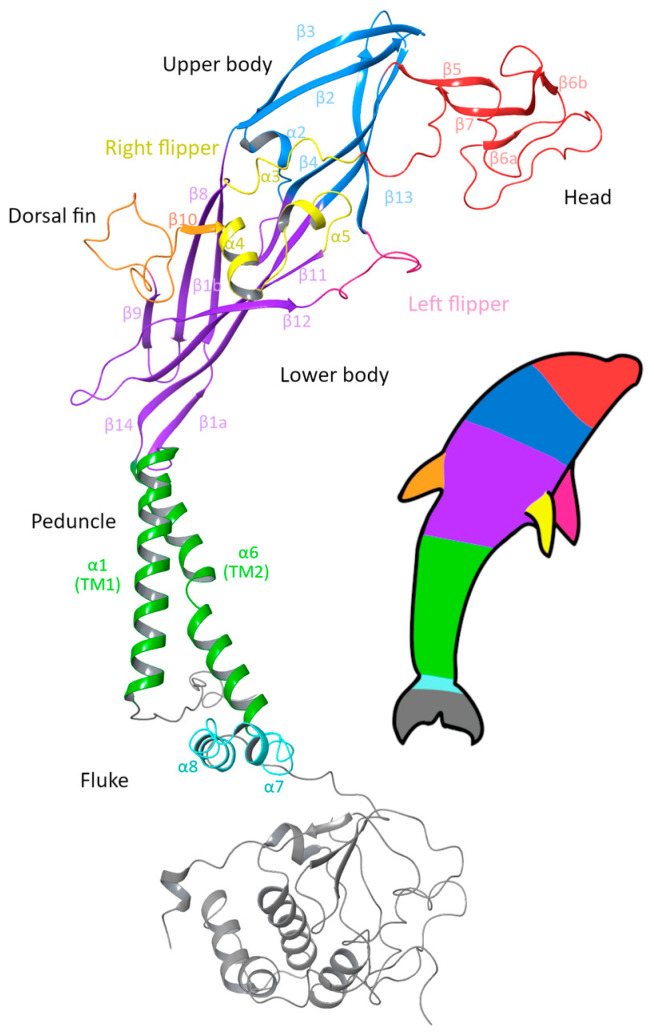
Ribbon diagram of a single subunit of the P2X7 receptor (P2X7A) with the updated dolphin representation to reflect the new structural findings of the intracellular domain. The head region (red), upper body (blue) and lower body (purple) form the extracellular domain, together with the dorsal fin (orange), right flipper (yellow) and left flipper (pink). The peduncle (green) corresponds to the two transmembrane helices, which link to the fluke corresponding to the C-cys anchor (cyan) and the cytoplasmic ballast (grey) that form the intracellular domain. Figure generated using Maestro 14.0 [44] with PDB ID 6U9W [39].

The P2X7R is composed of three subunits, which intertwine across both the extracellular and intracellular domains to form a trimeric receptor. The canonical (wild-type) human subunit is composed of 595 amino acids; beginning at the short intracellular N-terminal (M1–N25), the subunit contains two transmembrane (TM) domains, TM1 (Y26–V46) and TM2 (V335–I355), with a large extracellular domain between the two TM domains (S47–V334) and ends with a long intracellular C-terminal domain (D356–Y595). Visually, P2X subunits have been described as having a dolphin-like shape, consisting of a rigid body region with structurally flexible branching domains representing the head, dorsal fin, and left and right flippers of the dolphin [42,43]. In the original dolphin model that was developed from the structurally similar P2X4R, the tail represented the two transmembrane domains, and this description has remained for the P2X7R [34,43]. However, with the recent structures of the full-length rP2X7R being available, we suggest an updated P2X7R dolphin model (Figure 1) in which the body of the dolphin represents the extracellular domain, while the peduncle is the TM domain, and the fluke represents the large C-terminal domain.

### 2.2. Gating Mechanism

**Figure 2:**
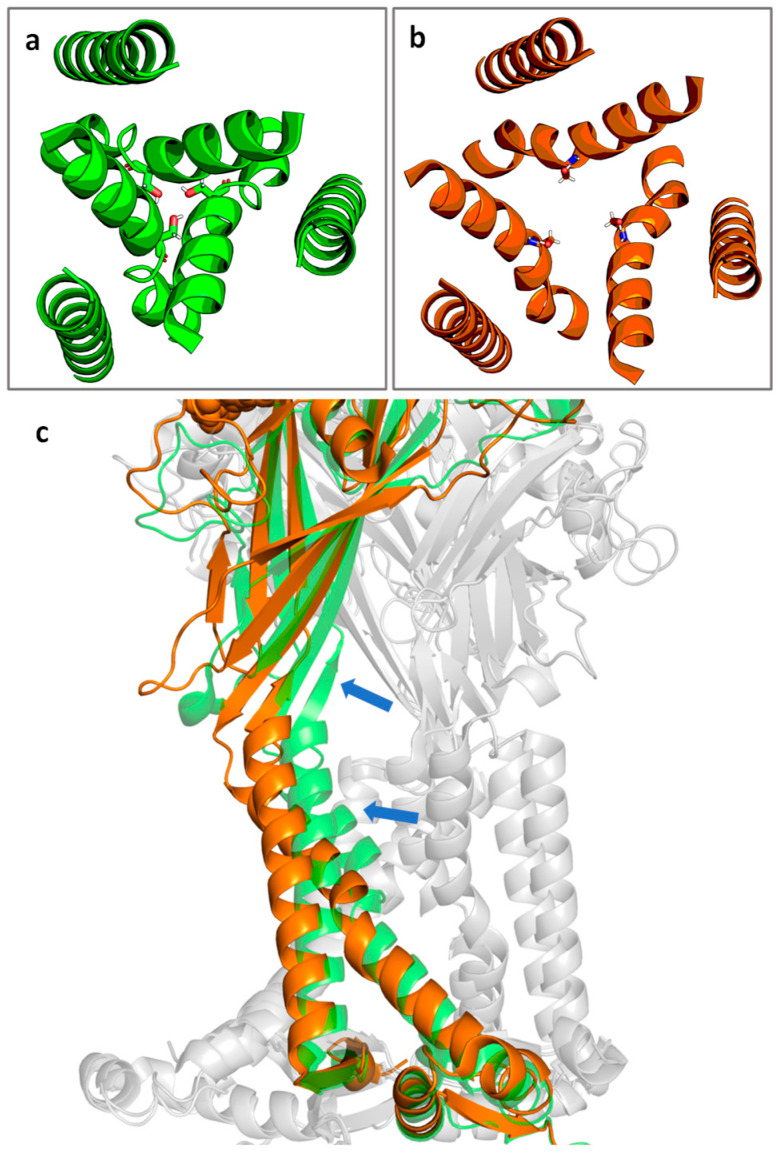
Cartoon representation of the top-down view of the P2X7R channel in the (**a**) closed state and (**b**) the ATP-bound open state with S342 shown in stick representation. (**c**) The transition from closed (green) to open (orange) state of a single subunit. Blue arrows indicate the approximate direction of motion. Non-highlighted subunits are shown in grey for context. Figure generated using PyMOL [45] with PDB IDs 6U9V (closed state) and 6U9W (open state) [39].

The P2X7R is gated by extracellular ATP. Channel opening results from movement of the TM gate which consists of residues S339 to S342, with the key residue S342 contributing to the narrowest part of the channel (Figure 2a,b) [39]. After agonist binding, the binding pocket undergoes a conformational change. The β strands of the extracellular domain flex outward, pulling the TM2 domains away from the channel lining and opening the channel (Figure 2c) [39].

In contrast to channel function, pore formation is poorly understood. Aspects of pore formation have been reviewed by North in 2002 [17], Di Virgilio et al. in 2018 [46], and Cevoli et al. in 2023 [47]. Functional studies often assess pore permeability using an assay that measures uptake of dyes or large cationic molecules, such as YOPRO-1 and ethidium, into the cell [48,49,50]. Pore formation can be reversible if ATP is quickly removed or degraded shortly after P2X7R activation, although this depends on cellular context [17,46,51]. However, prolonged activation often makes pore formation irreversible and eventually result in cell death, commonly due to necrosis from membrane disruption or apoptosis depending on context [17,46,52]. The mechanism underlying pore formation remains controversial. Currently, there are two hypotheses to explain this phenomenon. The first theory postulates that the receptor may itself become intrinsically permeable to large cations, supported by recent studies, but this remains a point of contention [46,53,54,55]. On the other hand, the second hypothesis suggests that receptor activation triggers a secondary protein such as pannexin-1 or other membrane hemichannels that interact with the P2X7R during prolonged activation [18,56]. Although it has been established that the P2X7R is involved in the activation of other proteins, it is unclear whether this results in the observed flux of large cations [57]. These two hypotheses are not mutually exclusive and both pathways may contribute to large cation transport.

## 3. The ATP Binding Site

**Figure 3:**
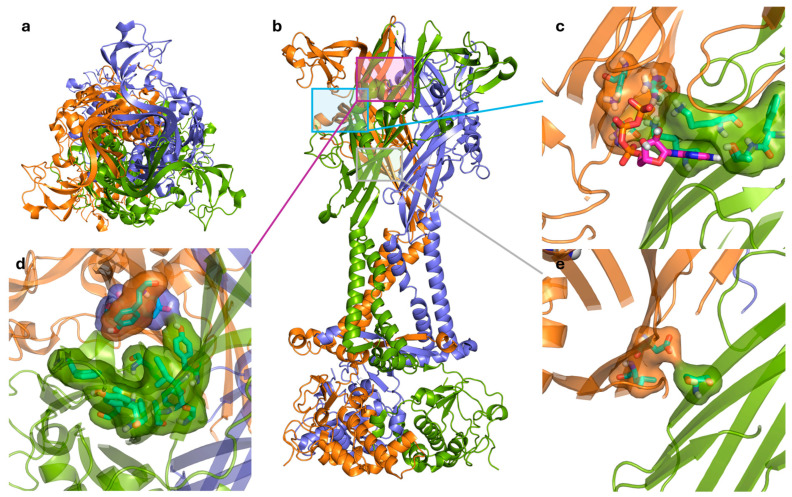
(**a**) Top view of the trimeric P2X7R in the open state showing the three subunits colored green, orange, and blue. (**b**) Side view of the receptor illustrating the transmembrane and extracellular domains, with boxed regions indicating the (**c**) ATP-binding site with the ATP molecule, the (**d**) central negative allosteric modulator binding site, and the (**e**) proposed positive allosteric modulator binding site. Ligands and interacting residues are shown in stick and surface representations, emphasizing key hydrogen bonds and hydrophobic contacts. Figure generated using PyMOL [45] with PDB IDs 6U9W (open state), 8TR5 (closed state), and 8TR6 (closed state with negative allosteric modulator A438079 bound) [39,40,41].

The ATP-binding site of the P2X7R has been reviewed by Jiang et al. in 2021 (Figure 3a–c) [58]. Before the determination of the receptor structure, comprehensive mutagenesis studies were conducted to identify key residues important for receptor activation [58]. By comparing known ATP-binding residues in other P2X receptors and studying highly conserved residues across different species, key residues in the hP2X7R were elucidated. The crystal structure of the zebrafish P2X4R (zfP2X4R) was used to identify eight key residues that form an inter-subunit binding site [43]. The location of this site is consistent with earlier mutagenesis studies that predicted an inter-subunit binding site [59,60]. The zfP2X4R and rP2X7R ATP-binding sites correspond to the following residues in hP2X7R: K64, K66, F188, and T189 from one subunit and N292, F293, R294, and K311 from the adjacent subunit (Figure 3c) [39,43,61]. In terms of pharmacology, ATP potency and efficacy differ between species and assays, with BzATP generally more potent than ATP at both receptors [62]. In dye uptake assays, which is a surrogate marker for pore formation, hP2X7R demonstrated greater sensitivity and greater dye uptake compared to the rP2X7R [62], which highlights a key species-specific difference. This difference is critical when using rodent models to study hP2X7R function and screening targeted therapies. Recent cryo-EM work identified that the hP2X7R has a species-specific interaction with ATP via residues L191, I214, and Y288 [31].

## 4. Allosteric Modulation and Binding Sites

### 4.1. Negative Allosteric Modulators

**Figure 4:**
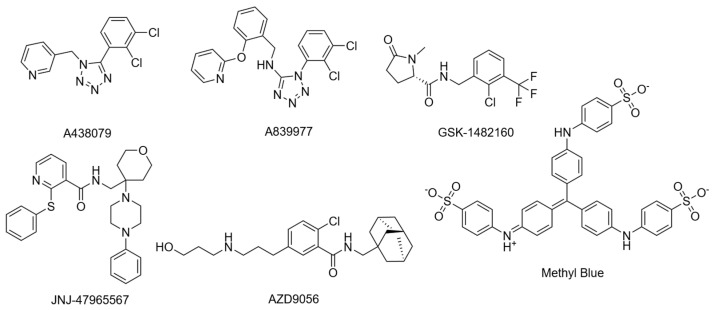
Structures of NAMs used to determine the NAM binding site.

Cryo-EM structures were recently resolved for the rP2X7R with six noncompetitive negative allosteric modulators (NAMs): A438079, A839977, AZD9056, GSK1482160, JNJ47965567, and methyl blue (Figure 4) [41]. The study observed an inter-subunit hydrophobic pocket that is distinct from the orthosteric ATP-binding site [41]. All structurally characterised NAMs bound to the inter-subunit hydrophobic pocket, consisting of residues F88, L95, F103, M105, Y108, F293, Y295, Y298, I310, and A312 of the rP2X7R (Figure 3d) [41]. Hydrogen bonding with residues D92 and K297 was also observed; however, this was highly variable between NAMs due to their chemical diversity [41]. The cryo-EM mapping of this pocket was in agreement with earlier mutational, functional, and structural studies, which identified amino acid residues involved in NAM binding [34,41,63]. Seven of the ten listed residues are conserved in the hP2X7R, with the three differing residues being F95, F108, and V312. Structural analysis showed that this hydrophobic pocket usually shrinks upon ATP binding, and NAMs prevent this conformational change, consequently inhibiting receptor function [34,41]. Importantly, the study by Oken et al. also noted that NAMs bind within the extracellular domain pocket, and direct contacts are absent in TM and intracellular domains [41]. There are some differences in antagonist characteristics between species. For example, the P2X7R antagonist Brilliant Blue G (BBG) is more potent in blocking calcium uptake in the rat receptor than the human receptor [62]. On the other hand, BBG can block the dye uptake of both species [62]. It was observed that residue F95 of the hP2X7R was important in forming pi-stacking interactions that the rP2X7R did not have [64]. However, they also noted that this change does not completely account for the differences in antagonistic effect, which warrants further investigation [64]. Cryo-EM studies suggest that V312 in the hP2X7R occupies more space, and alters the conformation of Y295, providing a structural basis for species-specific differences. These variations in allosteric sites are important to consider, as they can affect a drug’s translation to human studies. For a comprehensive structural analysis of these conformational states, readers are referred to the work by Oken et al. [31].

Cholesterol is another potential NAM that has a site distinct from the previous NAMs. Cholesterol and other steroid-like molecules have received little attention, and consequently their binding site is not known precisely. This presents a gap in research into the role of cholesterol inhibition on the P2X7R. Inhibition by cholesterol is dependent on a few factors, which are discussed in the section Membrane composition and receptor function.

### 4.2. Positive Allosteric Modulators

In contrast to NAMs, positive allosteric modulators (PAMs) have received relatively little attention despite their potential therapeutic applications and have been thoroughly reviewed by Stokes et al. [65]. PAMs can increase the effective or maximal response of a receptor (Type I), increase the receptor’s sensitivity to agonists (Type II), or a mixture of both (Mixed Type I/II). For the P2X7R, this leads to further activation of inflammatory or cell death pathways which may be beneficial for enhancing immune protection against pathogens [66], or for encouraging tumour death [65,67]. For instance, disease states where there are increased levels of extracellular ATP [68] or overexpression of the P2X7R [69] at affected sites may see therapeutic potential from PAMs. However, given the wide expression of the receptor, positive modulation is a challenge because activating P2X7R in non-diseased cells could drive inflammation or cytotoxicity, which may explain why PAMs remain understudied. Furthermore, the precise binding sites of many identified PAMs are unknown [70]. Among these, ginsenosides have been studied in greater detail using ligand docking and site directed mutagenesis to identify potential binding sites [70]. The proposed binding site of ginsenosides is distinct from that of NAMs, involving residues S60 of one subunit, and residues D318 and L320 of the neighbouring subunit [70]. This site is located between the lower body regions of two subunits and is only accessible when the P2X7R is in the open state (Figure 3e). Bidula et al. propose that PAM binding either stabilises the open state or facilitates the conformational changes associated with ATP binding [70]. Ivermectin is another recognised PAM and is thought to bind to the TM helices of the P2X4R [71,72]. Although primarily investigated in the P2X4R, ivermectin has shown activity in the hP2X7R, but not in rat or mouse P2X7Rs [65,73], and therefore species-specific interactions also complicate PAM development. Regardless, the widespread involvement of the P2X7R in disease states mean that PAMs have therapeutic potential, and their development still requires further research. Avoiding off-target effects via the use of antibody–drug conjugates and the potential for PAMs to restore function at loss-of-function mutant receptors have yet to be investigated. Alternatively, it may be beneficial to develop a P2X7R PAM to be used in conjunction with other cell-killing treatments. In this case, target-specific cell death is encouraged by further activation of the P2X7R, resulting in lower effective concentrations for both compounds. For example, a dysfunctional P2X7R variant that is chemotherapy-resistant (discussed in the section on P2X7B and alternative splicing heterotrimerisation) may become more susceptible to chemotherapy when receptor function is restored via PAM binding. Although variant-specific binding pockets have not yet been resolved, identifying these sites could guide drug design and the development of targeted modulators.

## 5. Membrane Composition and Receptor Function

The plasma membrane consists of many components in addition to the phospholipid bilayer, including lipids and proteins that enable essential cellular functions. It has been proposed that areas of the membrane can organise into domains enriched in sphingolipids and cholesterol, known as lipid rafts [74,75]. Lipid rafts act as platforms for signalling molecules and receptors to associate [76,77]. For a membrane protein such as the P2X7R, it is logical to consider how membrane composition, including cholesterol and lipid rafts, may affect receptor function.

### The Lipid Environment Critically Impacts P2X7R Activity

A study by Karasawa et al. on the panda P2X7R (pdP2X7) demonstrated the significance of membrane composition for P2X7R activity [55]. P2X7R constructs lacking either the N-terminal domain (ΔN, residues 1–22); the C-terminal domain (ΔC, from residue 360 onwards); and pdP2X7-ΔNC, a combination of the previous two constructs, were expressed in HEK293 cells and studied. These truncated receptors showed significantly reduced dye uptake, suggesting that these domains are important for pore formation in HEK293 cells [55].

However, when pdP2X7-ΔNC was reconstituted in liposomes composed of only 1-palmitoyl-2-oleoyl-sn-glycero-3-phosphoethanolamine (POPE) and 1-palmitoyl-2-oleoyl-sn-glycero-3-phospho-(1′-rac-glycerol) (POPG), both small cation and large dye uptake were preserved, suggesting that not one or the other terminal domain was strictly required for pore function in simplified lipid environments [55]. Subsequent experiments altered the lipid compositions of the liposomes, including POPE, POPG, 1-palmitoyl-2-oleoyl-sn-glycero-3-phosphocholine (POPC), 1-palmitoyl-2-oleoyl-sn-glycero-3-phospho-L-serine (POPS), sphingomyelin (SM) and cholesterol. These revealed that cholesterol has a dominant inhibitory role, while POPG and SM have a facilitatory role in dye uptake, and therefore pore formation [55]. Cholesterol inhibited pdP2X7-ΔNC in a dose-dependent manner, probably through directly interacting with the TM domain rather than indirect effects on bilayer rigidity [55]. A cholesterol depletion model using truncated receptor constructs was employed to identify the regions of the receptor that affected P2X7R’s sensitivity to membrane cholesterol. The pdP2X7-ΔC and pdP2X7-ΔNC constructs were not potentiated by cholesterol depletion, suggesting a loss of cholesterol sensitivity. In contrast, pdP2X7-ΔN had increased dye uptake upon cholesterol depletion, similar to the full-length receptor [55]. Notably, adding back the cysteine-rich region (C362 to C379) from the C-terminal domain to the pdP2X7-ΔNC construct restored cholesterol-mediated inhibition [55]. Mutation analysis then identified residues C362 and C363 as key (although not essential) residues in overcoming cholesterol-mediated inhibition [55]. The cysteine-rich region is located just beyond the second TM domain. Other studies in the past have shown cholesterol inhibition of the P2X7R [78] and the potential importance of the cysteine-rich region [78,79]. This region is subject to palmitoylation [55,80], a posttranslational modification in which fatty acids are covalently attached to cysteine residues. Cryo-EM structures and biochemical evidence suggest that palmitoylation anchors P2X7R to the membrane, stabilising the open state and preventing desensitisation, which is a key feature distinguishing the P2X7R from other P2X family members [39]. In addition, palmitoylation may promote association with lipid rafts, as a lack of palmitoylation reduces cell surface expression [80]. Interestingly, Karasawa et al. found similar surface expression of full-length pdP2X7R and truncated constructs in HEK293 cells [55], suggesting raft association rather than surface abundance may be the key determinate of modulating function. Finally, the recently resolved closed state structure of hP2X7R includes co-resolved cholesterol molecules, however their physiological importance remains uncertain [31]. Importantly, cholesterol has not been observed in P2X7R structures of other species.

Consequently, experimental and cellular membrane compositions must be considered when evaluating P2X7R activity. The association with lipid rafts is another factor to consider when studying different cells that will have their own lipid environments [78]. For example, truncated receptors lacking the cysteine-rich C-terminus may have reduced or no channel activity in cholesterol rich membranes (such as HEK293 cells), as a result of cholesterol inhibiting P2X7R function [55].

## 6. Genetic Variability: Single Nucleotide Polymorphisms of the P2X7R

### 6.1. SNPs Complicate Drug Development

Among the P2X family, the *P2RX7* gene is subject to the highest degree of genetic variation. Schäfer et al. found that when considering minor allele frequencies (MAFs) of at least 0.5%, the human *P2RX7* gene contained sixteen coding SNPs, while the rest of the P2X family collectively contained only seven [81]. As a consequence, *P2RX7* SNPs have received considerable attention.

In this review, we summarise the coding SNPs of the hP2X7R (Table 1 and Supplementary Table S1), aiming to highlight the complexity this creates for designing new drugs that therapeutically target the receptor. Although technically inaccurate, we refer to SNPs by their amino acid substitutions rather than nucleotide changes as this has more relevance to protein structure. Some SNPs are relatively frequent, and cause moderate changes in receptor function or expression, such as H155Y, R270H and A348T, which have MAFs > 25% [81,82]. The aforementioned SNPs tend to be well studied, however other rarer SNPs with more extreme effects on receptor function such as the SNP leading to R307Q—which causes a profound loss of channel and pore function [83]—have also been studied in great detail. Many SNPs show population-specific frequency differences, which complicates uniform therapeutic efficacy [81]. For instance, A348T has an overall MAF of approximately 31%, but ranges from 14% in the East Asian population to 46% in the African population [81]. Similarly, R270H MAF can range from 9% in the South Asian population to 40% in the East Asian population [81].

### 6.2. P2RX7 SNPs, Functional Effects and Disease Associations

*P2RX7* SNPs have been repeatedly reported to be associated with several disease states; however, such associations are usually population specific, and sometimes not replicated. For example, the E186K mutation, a loss-of-function SNP located close to the ATP-binding site, has been associated with hypertrophic cardiomyopathy, an inherited form of heart failure in young people [84]. The loss-of-function mutation R307Q in some cohorts has been associated with protection against development of multiple sclerosis [85]. V76A and I568N, both loss-of-function SNPs, have been associated with an increased risk of gout [86]. V76A has also been associated with another inflammatory condition, multiple sclerosis [87], although the same V76A appears to have a protective effect in sepsis and pneumonia [88]. This highlights the potential conflicting associations of SNPs.

H155Y and R270H are considered relatively weak gain-of-function SNPs [82,89] and have been associated with an increased risk of major depressive disorder (MDD) [90,91] and chronic pain [92]. Interestingly, R270H has also been associated with a decreased risk of chronic pain, which may have resulted from studying different pain types and phenotyping methods [93]. Functional studies suggest H155Y increases surface expression of the receptor rather than improvements in receptor function [94], although some studies vary [89]. In any case, synergism between H155Y, R270H, and A348T has been proposed [82]. A348T is located in the TM2 domain—the region critical for channel gating—which may explain its effect on P2X7R function [82,94]. A348T has been associated with an increased risk of gout [95] and a decreased risk of hepatocellular cancer [96]. The E496A substitution, which decreases function, has been associated with increased tuberculosis risk in two meta-analyses from 2010 and 2013 [97,98]. However, a more recent meta-analysis suggests that this association is limited to Asian populations [99].

### 6.3. P2RX7 SNPs in Mental Health Disorders

*P2RX7* SNPs have been extensively studied in mental health conditions, in particular MDD and bipolar disorder, as reviewed by Andrejew et al. [11], which was initiated by two pioneering genetic studies [100,101]. However, these links have been challenged. A meta-analysis conducted in 2014 found no association between Q460R and mood disorders [102], while another meta-analysis conducted in 2018 confirmed an association with MDD [103]. A 2019 study found no individual SNP was significantly associated with MDD but identified a risk-associated haplotype consisting of A348T~Q460R~rs1653625-A [104]. The last mutation, rs1653625 is a 3′ untranslated region (3′UTR) SNP that increased P2X7R expression by altering microRNA binding [104]. The link between *P2RX7* SNPs and bipolar disorder remains unconfirmed [101,103,105,106,107,108,109].

### 6.4. P2RX7 SNPs in Cancer

*P2RX7* missense mutations have been studied across a range of cancers, including pancreatic, papillary thyroid, breast, hepatocellular, and blood cancers [96,110,111,112,113,114,115]. Of these, chronic lymphocyte leukaemia (CLL) has been extensively studied [116]. E496A has been shown to increase the risk of CLL but has also been shown to improve the overall survival of CLL patients [113,114]. This difference could be attributed to variations in the role of the receptor at different stages of the disease [114]. However, other studies have failed to show an association between E496A and CLL [117,118,119], and a meta-analysis published in 2021 found no significant association between E496A and overall cancer risk [120]. The relevance of mutations in the risk of developing pancreatic cancer is also an interesting case, as studied by Magni et al. [110]. The study found two relevant mutations: G150R, causing a loss of channel and pore function, and R276H, which showed regular channel function but reduced pore function. While the G150R variants had a decreased risk of developing pancreatic cancer, R276H variants had an increased risk of developing pancreatic cancer [110]. This difference may be due to downstream signalling pathways, which are not well understood.

Although individual SNPs can confer significant changes in P2X7R function or expression, whether those changes lead to clinical effects, including symptoms or disease outcomes, depends on the specific biological or environmental context. Missense mutations can be associated with either protective or risk-increasing effects despite having similar effects on receptor function. This section focused on non-synonymous SNPs; however, synonymous SNPs have the potential to ‘silently’ alter protein expression and adds another aspect of variation to consider [121,122]. Additionally, SNPs located within introns should also be considered as they may give rise to spliced isoforms, as discussed in the section Splice Variants of the P2X7R.

**Table 1:** Non-synonymous SNPs in the hP2X7R that have been found to have disease associations, indicating their MAF, resultant amino acid change, and functional effects. Associations marked below are those reported in individual studies; several have negative or non-replicated results in other cohorts, as noted.

rsID	MAF *	Amino Acid Change	Functional Effect [Reference]	Disease Association [Reference]	Lack of Significant Disease Association ^#^ [Reference]
rs17525809	0.05 (0.02–0.07)	V76A	Partial loss of pore function [82] Partial loss of ion channel and pore function [123]	Decreased risk of pneumonia and sepsis [88] Increased risk of gout [86] and MS *^a^* [87]	Anxiety [124], IS *^b^* [125], MM *^c^* [126], pancreatic cancer [110]
rs28360447	0.01 (0.00–0.02)	G150R	Complete loss of pore function [82] Complete loss of ion channel and pore function [92,123] Complete loss of ion channel function and significantly reduced pore function [110]	Decreased risk of pancreatic cancer [110] Decreased BMD *^d^* [127]	MM *^c^* [126], schizophrenia [128]
rs208294	0.46 (0.35–0.71)	H155Y	Gain of pore function [82] Gain of ion channel function and increased rate of dye uptake [123] Gain of ion channel function but no significant effect on pore function [92]	Potential protective effect against Alzheimer’s disease [129] Increased risk of alcoholism [90], anxiety [90], chronic pain as PMP *^e^* [93], HHV-6A infection *^f^* [130], MDD *^g^* [90], SLE *^h^* with a history of pericarditis [131]	BMD *^d^* [132], chronic pain in OA *^i^* [93], IS *^b^* [125], MM *^c^* [126], PTC *^j^* [112], RA *^k^* [133], schizophrenia [128], SLE *^h^* [133], TB *^l^* [99]
rs28360451	<0.01 (<0.01)	E186K	Complete loss of ion channel and pore function [123]	Hypertrophic cardiomyopathy [84]	-
rs7958311	0.29 (0.05–0.45)	R270H	Loss of pore function [82] No significant change in pore function [123] Gain of ion channel function but loss of pore function [92]	Decreased risk of chronic pain [93] Increased risk of chronic pelvic pain [92], fibromyalgia [92], IBS *^m^* [92], MDD *^g^* with previous stress exposure [91]	Pancreatic cancer [110], TB *^l^* [99]
rs7958316	0.01 (0.00–0.02)	R276H	Complete loss of pore function [82] Normal channel function but reduced pore function [110] Loss of ion channel and pore function [92]	Increased risk of gout [86], pancreatic cancer [110]	Anxiety [124]
rs28360457	<0.01 (0.00–0.01)	R307Q	Complete loss of ion channel and pore function [83,92]	Decreased risk of MS *^a^* [85] Higher rate of bone loss in post-menopausal women [132,134] Increased risk of hepatocellular cancer [96]	BMD *^d^* [127], MM *^c^* [126], pancreatic cancer [110], RA *^k^* [133], schizophrenia [128], SLE *^h^* [133], disease severity in MS *^a^* [135]
rs1718119	0.31 (0.10–0.46)	A348T	Gain of ion channel and pore function [123] No significant effect on ion channel and pore function [92]	Decreased risk of hepatocellular cancer [96] Increased risk of gout [95], toxoplasmosis [136] Synergistic effect with Q460R, causing increased disease severity in relapse-remitting MS *^a^* [135]	Anxiety [124], BD *^n^* [107], chronic pain as PMP *^e^* and OA *^i^* [93], IS *^b^* [125], MDD *^g^* [104], MM *^g^* [126], pancreatic cancer [110], Schizophrenia [128], TB *^l^* [99]
rs2230911	0.14 (0.08–0.31)	T357S	Partial loss of ion channel and pore function [92,123]	Increased risk of pneumonia [88]	Hepatocellular cancer [96], MDD *^g^* [104], MM *^c^* [126], pancreatic cancer [110], RA *^k^* [133], Schizophrenia [128], SLE *^h^* [133], TB *^l^* [99], toxoplasmosis [136], disease severity in MS *^a^* [135]
rs2230912	0.07 (0.00–0.18)	Q460R	Partial loss of pore function [82] No effect [123] Gain of ion channel function but no effect on pore function [92]	Increased risk of anxiety [90], BD *^n^* development [101,105], MDD *^g^* [90,103] Synergistic effect with A348T, causing increased disease severity in relapse-remitting MS *^a^* [135]	BD *^n^* [106,107,108,109], chronic pain in PMP *^e^* and OA *^i^* [93], MDD *^g^* [102,106,107,108], MM *^c^* [126], ocular toxoplasmosis [136,137], pancreatic cancer [110], schizophrenia [128]
rs3751143	0.19 (0.10–0.29)	E496A	Partial loss of ion channel and pore function [92,123]	Decreased BMD *^d^* [127] Decreased risk of IS *^b^* [125] Increased risk of BD *^n^* [138], breast cancer [111], CLL *^o^* [113], follicular subtype of PTC *^j^* [112], hepatocellular cancer [96], ocular toxoplasmosis [137], Parkinson’s disease [139], TB *^l^* [97,98,140] Synergistic protective effect with H155Y against Alzheimer’s disease [129] Increased survival in CLL *^o^* [114]	Anxiety [124], BMD *^d^* [132], cancer [120], chronic pain in PMP *^e^* and OA *^i^* [93], CLL *^o^* [117,118], MDD *^g^* [104], MM *^c^* [126], pancreatic cancer [110], PTC *^j^* [112], RA *^k^* [133], Schizophrenia [128], SLE *^h^* [133], toxoplasmosis [136], disease severity in MS *^a^* [135]
rs1653624	0.01 (0.00–0.02)	I568N	Complete loss of ion channel and pore function [123] Partial loss of ion channel and pore function [92]	Higher rate of bone loss in post-menopausal women [134] Increased risk of gout [86]	BMD *^d^* [127,132], MM *^c^* [126], pancreatic cancer [110], schizophrenia [128]

* MAF: average minor allele frequency (Only >0.5% reported) in sample of 1000 Genomes Project, rounded to 2 decimal places [81]. Ranges are acquired from the Allele Frequency Aggregator (ALFA) [141] from dbSNP (https://www.ncbi.nlm.nih.gov/snp/docs/gsr/alfa/ (accessed on 16 April 2025)) [142,143,144]. ^#^ The list is not comprehensive; rather, it is intended to show that the lack of an association is still an important result to consider. *^a^* MS: multiple sclerosis, *^b^* IS: ischaemic stroke, *^c^* MM: multiple myeloma, *^d^* BMD: bone mineral density, *^e^* PMP: post-mastectomy, *^f^* HHV-6A: human herpesvirus 6A, *^g^* MDD: major depressive disorder, pain, *^h^* SLE: systemic lupus erythematosus, *^i^* OA: osteoarthritis, *^j^* PTC: papillary thyroid cancer, *^k^* RA: rheumatoid arthritis, *^l^* TB: tuberculosis, *^m^* IBS: irritable bowel syndrome, *^n^* BD: bipolar disorder, *^o^* CLL: chronic lymphocyte leukaemia.

## 7. Haplotypes and Functional Predictions

Rather than acting alone, SNPs often occur in haplotype blocks that co-influence P2X7R function by combining opposing or synergistic effects (e.g., gain and loss of function). Considering the frequency of *P2RX7* SNPs, a shift in focus from attributing disease to a single SNP toward a haplotype or haplotype pair may give clearer associations between P2X7R function and disease [82,134,145]. Jørgensen et al. have created a comprehensive resource of haplotypes detected in their study, and we have summarised these haplotypes together with the corresponding amino acid substitutions and frequencies (Table 2) [134]. The haplotype frequencies suggest that the most frequent haplotypes differ from the RefSeq wild-type haplotype (H) 2, which is in fact relatively rare, with a frequency of 5.3% (Table 2) [134]. Instead, there are multiple commonly expressed haplotypes, many of which contain two or more coding SNPs distinct from the wild type. In fact, this high degree of diversity has been observed previously in a different cohort; however, the haplotypes were grouped more broadly [82]. Although haplotypes have been identified, the resulting functions of some haplotypes have not been investigated or experimentally validated, and this remains as a gap in current research. The population-specific frequency differences in SNPs complicate the study of haplotypes. While the study by Jørgensen et al. is comprehensive, the dataset is constructed from a Danish cohort [134]. Haplotype frequencies may vary in different populations such as the South Asian or African population. Furthermore, certain populations may express haplotypes that are otherwise not present in other populations. For instance, the SNP P430R has an MAF of less than 1% in the European population, but greater than 10% in the African population [141]. More work is needed to identify the variability of haplotype frequencies between populations.

### Haplotypes May Better Explain Disease Associations

The consideration of haplotypes is important, as it avoids the confounding effects that would otherwise be present when analysing individual SNPs. This is especially so for the hP2X7R, which has a degree of polymorphism such that most individuals are expected to co-express two different copies of the hP2X7R [81]. For instance, a patient expressing P2X7-H9 would present with the loss-of-function V76A and gain-of-function A348T substitutions. The overall function of the receptor is predicted to increase [134], but the patient would still be considered a part of V76A mutants during genetic analyses. A more comprehensive example in a similar situation can be seen in P2X7-H14 and P2X7-H15. While Q460R confers a relatively small change in function, the variant is also likely to contain an A348T mutation, which causes an increase in channel and pore function. The overall result is the expression of a receptor with increased channel and pore function, leading to elevated secretion of the proinflammatory cytokine IL-1β [82]. Without considering haplotypes, Q460R may be attributed to disease states when it is more accurately described as part of the P2X7-H14 and P2X7-H15 haplotypes.

Can haplotype analysis translate to a disease association? This was hinted at previously, where a haplotype consisting of A348T~Q460R~rs1653625-A, corresponding to P2X7-H14/H15, was associated with increased MDD severity [104]. A more recent study conducted by Guerini et al. in 2022 supports this sentiment, showing that a haplotype corresponding to P2X7-H14/H15 resulted in increased disease severity in relapse-remitting multiple sclerosis, while the complementary haplotype (A348 and Q460) was shown to be protective against disease severity [135]. Haplotype-based analysis may provide clearer associations with disease phenotypes and should be prioritised in future research and clinical design. Similar with disease associations in SNPs, haplotype associations will require replication, especially in different populations, where large variations in SNPs will also likely lead to variations in expressed haplotypes.

## 8. Splice Variants and Alternatively Spliced Heterotrimers

### 8.1. Splice Variants

Alternative splicing is another important consideration for the P2X7R, as it can lead to deletion or inclusion of whole exons, resulting in the expression of subunits that are distinct from the wild type. Multiple splice variants of P2X7R exist and these variants can form homotrimers or heterotrimers with wild-type subunits, sometimes enhancing or reducing functionality. Their roles in disease progression and therapeutic resistance, such as in leukaemia, are underexplored.

The alternatively spliced isoforms have been reviewed [38], and their structures predicted by AlphaFold [148]. The nomenclature used to refer to splice variants in this review follows that of Cheewatrakoolpong et al. who employed alphabetical ordering to distinguish variants (Table 3) [149]. The study compared splice variants P2X7B and P2X7H against the wild type, P2X7A, in terms of expression, ion channel function, and pore formation [149]. P2X7B lacks the long C-terminus of the receptor (Δ-C) that has been replaced by a shortened sequence, while P2X7H lacks the TM1 domain (ΔTM-1) [149]. P2X7B was found to retain ion channel function; however, it reduced agonist sensitivity and resulted in a significant impairment in pore formation [149]. In contrast, P2X7H showed no activity in response to the agonist BzATP, suggesting that P2X7H is a non-functional receptor. Interestingly, P2X7B has been reported as highly expressed in some tissues and may represent a predominant isoform in some physiological contexts [149].

If cells expressing the P2X7R do not exhibit pore formation, it is advisable to assess which isoforms are being expressed before drawing conclusions. Other variants (C, D, E, F, and G) could not to be analysed at the time due to difficulties developing appropriate reagents to perform gene expression studies [149]. P2X7E was later characterised as a non-functional receptor [150]. Additionally, other variants have been identified, but most of these remain uncharacterised, presenting a gap in current research.

**Table 3:** Splice variants of the hP2X7R, their functional differences, and Genbank accession number unless otherwise stated [151,152]. The variant P2X7I results in a null allele [153]. P2X7K has been identified in the rat and mouse but not in the human [154].

Variant Name	Suggested Effects [Reference] ^a^	NCBI Accession Number
P2X7A	Normal function [149,155]	Y09561.1 (RefSeq: NM_002562.6)
P2X7B	Reduced agonist sensitivity, but similar channel function to P2X7A homotrimers [149,156] Significant reduction in pore formation [149,156] Little change to antagonist sensitivity [149,156] Heterotrimer formation with P2X7A [156]	AY847298.1
P2X7C	N/I [149]	AY847299.1
P2X7D	N/I [149]	AY847300.1
P2X7E	Deletion of ATP-binding site No surface expression, leading to a non-functional receptor [149,150]	AY847301.1
P2X7F	N/I [149]	AY847302.1
P2X7G	N/I [149]	AY847303.1
P2X7H	Non-functional receptor [149]	AY847304.1
P2X7J	Deficient pore formation Reduced channel function Heterotrimer formation with P2X7A [155]	DQ399293.1
P2X7L	Loss of channel and pore function due to deletion of ATP-binding site Heterotrimer formation with P2X7A [150]	MK465687.1
P2X7M ^b^ (ΔE2)	N/I [89]	-
P2X7N	N/I [150]	MK465688.1
P2X7O	N/I [150]	MK465689.1
P2X7P	N/I [150]	MK465690.1
P2X7Q	N/I [150]	MK465691.1
Variant 4 (V4)	N/I [157]	- (RefSeq [146]: NR_033950.2)
Variant 7 (V7)	N/I [157]	- (RefSeq [146]: NR_033953.2)

^a^ N/I = not investigated, ^b^ as suggested by De Salis et al. [38].

### 8.2. P2X7B and Alternative Splicing Heterotrimerisation

An added complication is that of heterotrimerisation. Heteromers, or heterotrimers for the P2X7R may refer to the formation of P2X7Rs composed of different isoforms or as receptors formed between different P2X subunits. We will use the term alternatively spliced (AS) heterotrimers to distinguish the two forms. For example, mixtures of P2X7A and P2X7B isoforms can associate in four different ways—homotrimeric forms P2X7A and P2X7B, and AS heterotrimeric forms P2X7A/(P2X7B)_2_ and (P2X7A)_2_/P2X7B. A study analysed P2X7B in detail and found similar changes in terms of expression, ion channel function, agonist response, and pore formation [156]. However, the study also considers AS heterotrimeric constructs of P2X7A and P2X7B, finding that heterotrimers can form between the variants, called P2X7A/(P2X7B)_2_ and (P2X7A)_2_/P2X7B heterotrimers. Unexpectedly, preliminary data suggested these heterotrimers may exhibit increased pore formation compared to homotrimers of P2X7A and P2X7B, though this remains as preliminary evidence requiring replication [156]. The study hypothesised that P2X7B was better for growth promotion compared to P2X7A, since channel function was retained, but pore formation, which has been linked to cell cytotoxicity, had been abolished [156]. Therefore, it was suggested that the cell’s response to extracellular ATP depended on the expression of P2X7A and P2X7B, noting that this dichotomy is unique to the P2X7R [156].

Another study found that the expression of P2X7B is linked to chemotherapy resistance and relapse in acute myeloid leukaemia (AML) patients overexpressing P2X7B [158]. The chemotherapeutic agent daunorubicin (DNR) causes cell death and the release of ATP into the extracellular microenvironment. AML cells expressing high levels of either P2X7A or P2X7B were treated with DNR and it was found that DNR toxicity was increased in cells with high P2X7A expression, while cells expressing high levels of P2X7B were protected from DNR-dependent death [158]. Furthermore, when DNR was combined with the P2X7R NAM AZ10606120 in an in vivo mouse model, it was found that coadministration was more efficacious than single treatment using either agent in reducing leukaemia growth [158]. Treatment with DNR kills cells with a higher expression of P2X7A, while application of a P2X7R inhibitor blocks cell proliferation induced by the DNR-resistant P2X7B [158]. This indicates the therapeutic potential of modulating the P2X7R as well as the contribution of splice variant expression in disease states.

### 8.3. Splice Variants Are Not Well Characterised

The formation of heterotrimers between P2X7 splice variants has not been widely studied. A major barrier to investigating AS heterotrimers is the difficulty in detecting and distinguishing different splice variants. Without the ability to detect the different AS heterotrimers, it is difficult to understand their expression and physiological effects compared to the receptor made up of three wild-type subunits. Additionally, the relevance of isoforms and AS heterotrimers in disease states remain largely unexplored. The existence of at least 17 human splice variants, some of which have not been studied in detail, suggests that there are many possible heterotrimeric P2X7R combinations with unknown physiological roles. In the context of drug development, attempting to design a modulator to inhibit pore formation may be futile if pore formation is absent. Conversely, heterotrimers with increased agonist sensitivity or pore formation may have therapeutic potential, depending on their pattern of expression.

A significant obstacle to this research is the lack of in vitro techniques to control receptor stoichiometry when expressing splice forms in model systems. Other fields, such as GABA_A_ and nicotinic acetylcholine receptor research, have benefitted from techniques that concatenate subunits, enabling control over receptor assembly [159,160]. Concatenation studies have not yet been conducted between P2X7 splice variants. Alternatively, deep learning models such as AlphaFold have enabled the modelling of heterotrimeric assemblies of P2X7R [148]. When molecular dynamic simulations were used to assess these generated models, the heterotrimers were found to be structurally stable [148]. However, further experimental and computational studies are needed to support these predictions and the structural and functional outcomes of splice variants and heterotrimers.

## 9. Clinical Failures and Therapeutic Barriers

### 9.1. Clinical Trials and the Polymorphous P2X7R

**Figure 5:**
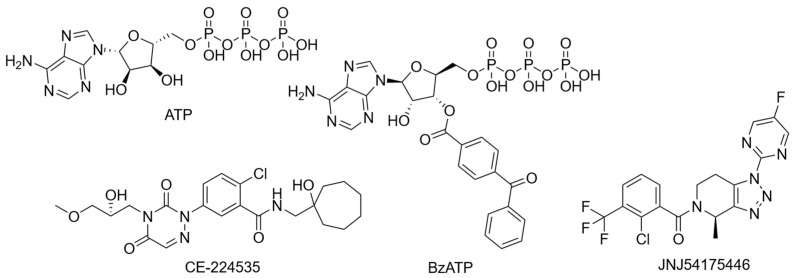
Structures of frequently used agonists (ATP and BzATP) as well as antagonists used in clinical trials.

The preceding sections highlight the individual variability of the P2X7R and its subunits, but in combination, this variability underscores the complexity of studying the receptor. Despite strong preclinical rationale, several P2X7R antagonists have been tested, and all have failed to prove efficacy in clinical trials. Two benzamide antagonists, AZD9056 (Figure 4) and CE-224,535 (Figure 5), have been evaluated in phase IIa and IIb randomised clinical trials for their efficacy in rheumatoid arthritis resistant to conventional treatments [161,162]. Both compounds failed to demonstrate significant efficacy compared to placebo [161,162]. More specifically, AZD9056 was shown to be effective in a phase IIa trial, with significant improvements in markers of rheumatoid arthritis severity: however, this did not translate in a phase IIb trial [161]. Clinical failure was attributed to the complex pathogenesis of the condition, suggesting P2X7R antagonism alone may be insufficient to manage inflammation [161,162]. AZD9056 was also evaluated as ineffective in a phase IIa randomised clinical trial in patients with moderately to severely active Crohn’s disease [163]. While more patients treated with AZD9056 responded to treatment positively compared to placebo, the difference was not statistically significant [163]. Another placebo-controlled, randomised clinical trial was conducted to assess the safety and tolerability of the triazolopyridine P2X7R antagonist JNJ-54175446 (Figure 5) in patients with MDD and under acute sleep deprivation, serving as a proxy for aggravated neuroinflammation [164]. Although JNJ-54175446 was found to show targeted engagement and was safe and tolerable, clinical efficacy on mood remains inconclusive [164]. Despite proving pharmacological activity in ex vivo models [164,165], levels of IL-1β in peripheral blood after JNJ-57417446 remain unchanged compared to placebo, suggesting other potential pathways leading to IL-1β release [164].

Variability in SNPs, splice variant expression, and membrane composition likely underlies inconsistent responses. While no mutations can be clearly linked to the disease states studied in P2X7 clinical trials, none of these trials conducted subgroup analyses based on the genetic background of the study populations. Furthermore, testing was not performed to confirm the receptor’s expression or functionality, including calcium flux or dye uptake. Drugs targeting the channel or pore may be ineffective in receptors already compromised by mutation. Therefore, genotyping and isoform profiling should be standard in clinical studies of P2X7Rs. Secondary markers of activity such as IL-1β levels may also be informative but may not be representative of P2X7R function. Stratifying the population by receptor function and genotypes will allow for more direct links between a compound and its efficacy. A demonstration of this can be seen in a phase I clinical trial that tests topical non-functional P2X7 (nfP2X7)-targeted antibodies for the treatment of basal cell carcinoma [166]. While it is too early to determine efficacy, target specificity appears to play an important role, especially so for the variable P2X7R.

The frequencies of SNPs can vary significantly between ethnicities, complicating the development of universally effective therapeutics [81]. For example, NAMs that inhibit channel function and pore formation may have reduced efficacy in receptors with SNPs that already impair these functions. While individual SNPs can significantly alter P2X7R function, their correlation with disease risk, such as MDD or bipolar disorder, can remain unclear. Focusing on haplotypes or haplotype pairs may give clearer associations between P2X7R function and disease, though this area is still relatively understudied. This same recommendation applies to clinical studies, where participant genotyping should be performed prior to enrolling in trials. In addition, splice variant heterotrimers will also alter outcomes, and there remains a pressing need to further explore how alternative splicing is regulated in different disease states and environments.

### 9.2. The P2X7R Has Potential as a Diagnostic or Prognostic Marker

A lot of attention has been focused on the P2X7R as a drug target; however, the receptor’s expression can also be used as a biomarker. In COVID-19, increased levels of detectable P2X7Rs served as markers of disease severity [15] and unfavourable clinical outcomes [167]. An in vitro model of tuberculosis showed that P2X7R expression levels distinctly worsened the outcome of severe forms of tuberculosis [168]. In cancers, nfP2X7 have been shown to be useful as an early diagnostic marker [169] and as a prognostic marker [22,170,171]. Several brain-penetrant radio-labelled ligands have been developed and used in human studies, highlighting the receptor’s potential as a biomarker [172,173,174,175]. For instance, these radio-labelled ligands have been used to highlight the P2X7R as a biomarker for epilepsy [176]. While P2X7R expression is promising as a biomarker, the impact of receptor mutations remains comparatively unexplored, likely due to challenges in isoform identification and the lack of haplotype studies.

## 10. Forward-Looking: Potential of In Silico Studies in the P2X7R

### New Structural Insights Now Enable Computational Investigations

Structural determination of the rP2X7R has resolved the TM and intracellular domains of the receptor that were previously uncharacterised [39]. Further cryo-EM studies have produced full-length cryo-EM structures at higher resolutions, enabling identification of structural water molecules, particularly within the TM domain, and suggesting a bound sodium ion in the channel [40,41]. Several agonists and antagonists have also been co-resolved with the rP2X7R (Table 4) [39,40,41]. These structures will provide a good foundation for in silico studies, although some regions remain unresolved that will require additional modelling. For most structures, the missing regions include the N-terminus (M1–C5), a loop in the upper body (N74–T81), and a large intracellular loop (S443–R471). Additionally, structures of splice variants of the receptor have been predicted through deep learning methods, presenting opportunities for further study [148].

In silico techniques such as ligand docking and molecular dynamics simulations will greatly benefit from the newly resolved P2X7R structures. These advances will enable the modelling of features of the receptor that have not been able to be explored, including splice variants, functional dependence on membrane composition, and the effects of SNPs on binding and receptor conformation. For example, SNPs resulting in E186K and L191P, both located near the ATP-binding site, may affect ATP binding and loss of P2X7R function [84,123]. Similarly, the R276H mutation, located close to the PAM binding site, abolishes P2X7R-dependent dye uptake [82]. Furthermore, splice variants can be computationally assembled as AS homo- or heterotrimers, allowing the modelling of stoichiometries that are difficult to resolve experimentally. Complex conditions involving combinations of SNPs and AS heterotrimers can now be simulated, making it possible to study the resultant conformational changes. Given the extensive in vitro and in vivo data now available, along with multiple resolved P2X7R structures, in silico approaches are highly encouraged to support and refine future studies. Although a few studies have explored P2X7R using molecular docking [63,70,181] and molecular dynamics simulations [31,181], these have mainly focused on analysing ligand binding. Similar computational approaches in other receptors have defined membrane cholesterol interactions [182,183], effects of mutations on protein structure [184], and mechanistic insights of ligand binding [185], showing the value of such techniques in P2X7R research.

Historically, many studies have been conducted on the non-human receptor. With current modelling techniques, a homology model of the hP2X7R can be generated using the cryo-EM structure as a template. Notably, rat and hP2X7Rs share approximately 80% identity and conserve key residues, supporting the validity of homology modelling, although subtle species-specific pharmacological differences remain.

## 11. Conclusions

The polymorphic nature of the P2X7R and splice isoform variants has likely contributed to the clinical failure of antagonists. A detailed understanding of receptor biology, including genetic, structural, and membrane influences, is essential for therapeutic progress. In silico tools and genotyped clinical trials represent the next steps toward realising the therapeutic potential of the P2X7R.

This review has outlined the mutations of the P2X7R and their functional implications. Without a comprehensive understanding of the receptor’s genetic variability, developing agents to alter the function of this receptor will remain challenging. Considering the large number of SNPs that alter the function of the P2X7R, the presence of the less-sensitive splice variant P2X7B, and various haplotype combinations, it is important to account for these genetic factors when designing compounds and planning clinical trials. Drug development efforts should consider binding modes in the context of SNPs that affect ligand binding. Future clinical trials would benefit from genotyping participants and/or measuring isoform expression to enable stratified analyses based on the predicted P2X7R function. Moreover, understanding how antagonists interact with AS heterotrimers is critical, but the identification of splice variants and heterotrimers remains a large gap in the field. In silico techniques have the potential to resolve heterotrimeric structures, but they remain underutilised. Although the P2X7R is a highly attractive target, many characteristics remain poorly understood, which likely contributes to the lack of clinical success, to date.

## Figures and Tables

**Table 2:** P2X7 variants derived from common haplotype blocks as determined by Jørgensen et al. [134]. The table is organised with respect to the 4-SNP haplotype block predicted by Stokes et al., involving residues A348, T357, Q460, and E496 [82]. The wild-type H2 has been shaded grey and refers to the base sequence described in RefSeq Accession number NM_002562.6 [146,147]. Bolded residues highlight differences from the wild-type H2.

Subtype	Frequency (%)	Amino Acid (Nucleotide) Position and Residue										
		76 (253)	150 (474)	155 (489)	270 (835)	276 (853)	307 (946)	348 (1068)	357 (1096)	460 (1405)	496 (1513)	568 (1729)
H1	16.20	Val	Gly	His	**His**	Arg	Arg	Ala	Thr	Gln	Glu	Ile
H2	5.29	Val	Gly	His	Arg	Arg	Arg	Ala	Thr	Gln	Glu	Ile
H3	4.43	Val	Gly	**Tyr**	**His**	Arg	Arg	Ala	Thr	Gln	Glu	Ile
H4	2.57	Val	Gly	His	Arg	Arg	Arg	Ala	Thr	Gln	Glu	**Asn**
H5	2.31	Val	Gly	**Tyr**	Arg	Arg	Arg	Ala	Thr	Gln	Glu	Ile
H6	1.40	Val	Gly	**Tyr**	**His**	**His**	Arg	Ala	Thr	Gln	Glu	Ile
H7	1.06	Val	Gly	His	**His**	Arg	**Gln**	Ala	Thr	Gln	Glu	Ile
H8	15.34	Val	Gly	His	Arg	Arg	Arg	**Thr**	Thr	Gln	Glu	Ile
H9	4.54	**Ala**	Gly	His	Arg	Arg	Arg	**Thr**	Thr	Gln	Glu	Ile
H10	2.40	Val	Gly	**Tyr**	Arg	Arg	Arg	**Thr**	Thr	Gln	Glu	Ile
H11	11.77	Val	Gly	**Tyr**	Arg	Arg	Arg	Ala	Thr	Gln	**Ala**	Ile
H12	2.57	Val	Gly	His	Arg	Arg	Arg	Ala	Thr	Gln	**Ala**	Ile
H13	1.03	Val	**Arg**	**Tyr**	Arg	Arg	Arg	Ala	Thr	Gln	**Ala**	Ile
H14	13.94	Val	Gly	**Tyr**	Arg	Arg	Arg	**Thr**	Thr	**Arg**	Glu	Ile
H15	1.23	Val	Gly	His	Arg	Arg	Arg	**Thr**	Thr	**Arg**	Glu	Ile
H16	5.00	Val	Gly	His	Arg	Arg	Arg	Ala	**Ser**	Gln	Glu	Ile
H17	3.43	Val	Gly	**Tyr**	Arg	Arg	Arg	Ala	**Ser**	Gln	Glu	Ile

**Table 4:** Available structures of the P2X7R from PDB (http://www.rcsb.org/ (accessed on 25 March 2025)) [177] and their sequence identity to the hP2X7R determined by BLAST (https://blast.ncbi.nlm.nih.gov/Blast.cgi (accessed on 16 April 2025)) [178]. Resolution has been rounded to 1 decimal place.

Species	Sequence Identity to Human (%)	Technique Used	PDB ID [Reference]	Resolution (Å)	Bound Ligand	Pharmacodynamic Class
Chicken	45	X-ray	5XW6 [179]	3.1	TNP-ATP	Competitive antagonist
Panda	85	X-ray	5U1L [34]	3.4	-	Apo state
			5U1U [34]	3.6	A740003	Allosteric antagonist
			5U1V [34]	3.4	A804598	Allosteric antagonist
			5U1W [34]	3.5	AZ10606120	Allosteric antagonist
			5U1X [34]	3.2	JNJ47965567	Allosteric antagonist
			5U1Y [34]	3.3	GW791343	Allosteric antagonist
			5U2H [34]	3.9	ATP A804598	Agonist Allosteric antagonist
		Cryo-EM	8JV7 [180]	3.6	PPADS	Competitive antagonist
			8JV8 [180]	3.3	PPNDS	Competitive antagonist
			8Z1D	4	PSFL1191	Allosteric antagonist
			8Z0Z	3.3	JNJ-54175446	Allosteric antagonist
Mouse	81	Cryo-EM	9E3Q *^a^* [31]	2.5	-	Apo state
Rat	80	Cryo-EM	6U9V *^a^* [39]	2.9	-	Apo state
			6U9W *^a^* [39]	3.3	ATP	Agonist
			8TR5 *^a^* [40]	2.5	-	Apo state
			8TR6 *^a^* [41]	2.2	A438079	Allosteric antagonist
			8TR7 *^a^* [41]	2.5	A839977	Allosteric antagonist
			8TR8 *^a^* [41]	2.2	AZD9056	Allosteric antagonist
			8TRA *^a^* [41]	2.4	GSK1482160	Allosteric antagonist
			8TRB *^a^* [41]	2.4	JNJ47965567	Allosteric antagonist
			8TRJ *^a^* [40]	2.8	BzATP	Agonist
			8TRK *^a^* [41]	2.7	Methyl blue	Allosteric antagonist
			8V4S *^a^* [40]	2.5	-	Apo state
Human	100	Cryo-EM	9E3M *^a^* [31]	2.5	-	Apo state
			9E3N *^a^* [31]	3.0	ATP	Agonist
			9E3O *^a^* [31]	2.8	UB-ALT-P30	Allosteric antagonist
			9E3P *^a^* [31]	2.5	UB-MBX-46	Allosteric antagonist

*^a^* Resolved structure includes the transmembrane and intracellular domain.

## Data Availability

No new data were created or analyzed in this study. Data sharing is not applicable to this article.

## Supplementary Materials

The following supporting information can be downloaded at https://www.mdpi.com/article/10.3390/ijms262110265/s1.
