# Supplementary material for: Genetic Variations in the P2X7 Receptor: Opportunities and Challenges for Drug Development
Source: Int J Mol Sci. 2025 Oct 22;26(21):10265. doi: 10.3390/ijms262110265 (PMC12607368; doi:10.3390/ijms262110265)
Supplement: Supplementary file 1 [file ijms-26-10265-s001.zip › ijms-3911553-supplementary.pdf]

Supplementary Information

Genetic Variations of the P2X7 Receptor: Opportunities and Challenges for Drug Development

Justin Cheah[1,2], Kristen K. Skarratt[3], Stephen J. Fuller[3], Thomas Balle[1,2]

[1]Sydney Pharmacy School, Faculty of Medicine and Health, The University of Sydney, Sydney, NSW 2006, Australia

[2]Brain and Mind Centre, The University of Sydney, Camperdown, NSW 2050, Australia

[3]Sydney Medical School Nepean, Faculty of Medicine and Health, The University of Sydney, Nepean Hospital, Penrith, NSW 2750, Australia

[Corresponding authors] Thomas Balle and Stephen Fuller

Justin Cheah

justin.cheah@sydney.edu.au

https://orcid.org/0009-0008-0690-7566

Kristen K. Skarratt

kristy.skarratt@sydney.edu.au

https://orcid.org/0000-0003-4971-2773

Thomas Balle

thomas.balle@sydney.edu.au

https://orcid.org/0000-0002-0233-8350

Stephen J. Fuller

stephen.fuller@sydney.edu.au

https://orcid.org/0000-0003-4637-7391

Supplementary Table S1. Non-synonymous SNPs of the hP2X7R that have no identified disease association, indicating their MAF, resultant amino acid change and functional effects.

rsID	MAF*	Amino acid change	Functional effect [Reference]
-	-	V80M	No effect [89]
rs28360445	< 0.01 (< 0.01)	R117W	Partial loss of ion channel and pore function [123]
-	-	A166G	Gain of pore function [89]
rs28360452	< 0.01 (< 0.01)	L191P	Partial loss of ion channel and pore function [123]
rs74357548	0.011 (0.00-0.03)	D423N	No effect on ion channel function, but partial loss of pore function [92]
rs10160951	0.036 (0.00-0.23)	P430R	Gain of ion channel function, but normal pore function [92]
rs28360459	0.029 (0.00-0.12)	A433V	No effect [92]
rs2230913	0.04 (0.00-0.04)	H521Q	No effect [92, 123]

*MAF: Average minor allele frequency (Only >0.5% reported) in sample of 1,000 Genomes Project [81]. Ranges are acquired from the Allele Frequency Aggregator (ALFA) [141] from dbSNP (https://www.ncbi.nlm.nih.gov/snp/docs/gsr/alfa/) [142-144]
